# Nanosecond pulsed electric fields modulate the expression of the astaxanthin biosynthesis genes *psy*, *crtR-b* and *bkt 1* in *Haematococcus pluvialis*

**DOI:** 10.1038/s41598-020-72479-5

**Published:** 2020-09-23

**Authors:** Fan Bai, Christian Gusbeth, Wolfgang Frey, Peter Nick

**Affiliations:** 1 Botanical Institute, Karlsruhe Institute of Technology, Fritz-Haber-Weg 4, 76131 Karlsruhe, Germany; 2 Institute for Pulsed Power and Microwave Technology (IHM), Karlsruhe Institute of Technology, Campus Nord, 76344 Eggenstein-Leopoldshafen, Germany

**Keywords:** Biological techniques, Cell biology, Plant sciences, Engineering, Nanoscience and technology

## Abstract

Nanosecond pulsed electric fields (nsPEFs) have been extensively studied with respect to cellular responses. Whether nsPEFs can regulate gene expression and to modulate the synthesis of valuable compounds, has so far been only tested in the context of apoptosis in cancer cells. We used the unicellular algae *Haematococcus pluvialis* as system to test, whether nsPEFs could alter gene expression and to promote the biosynthesis of astaxanthin. We find that nsPEFs induce a mild, but significant increase of mortality up to about 20%, accompanied by a moderate increase of astaxanthin accumulation. Steady-state transcript levels of three key genes *psy*, *crtR-b* and *bkt 1* were seen to increase with a maximum at 3 d after PEF treatment at 50 ns. Pulsing at 25 ns reduce the transcripts of *psy*, *crtR-b* from around day 2 after the pulse, while those of *bkt 1* remain unchanged. By blocking the membrane-located NADPH oxidase RboH, diphenylene iodonium by itself increased both, the levels of astaxanthin and transcripts of all three biosynthetic genes, and this increase was added up to that produced by nsPEFs. Artificial calcium influx by an ionophore did not induce major changes in the accumulation of astaxanthin, nor in the transcript levels, but amplified the response of *crtR-b* to nsPEFs at 25 ns, while decreased in 50 ns treatment. When Ca^2+^ influx was inhibited by GdCl_3_, the transcript of *psy* and *bkt 1* were decreased for both 25 ns and 50 ns treatments, while *crtR-b* exhibited an obvious increase for the 25 ns treatment. We interpret these data in a working model, where nsPEFs permeabilise plasma and chloroplast membrane depending on pulse duration leading to a differential release of plastid retrograde signaling to the nucleus.

## Introduction

Nanosecond pulsed electric fields (nsPEFs) were originally developed as a technology to induce electroporation on cell plasma membrane to promote drug delivery, especially to interior organs. The combination of high field strength (up to 300 kV·cm^−1^) and extremely short duration of pulses (ns-range) was thought to circumvent the otherwise irreversible permeabilisation of the membrane that often accompanies conventional electroporation. Subsequently to the nsPEFs treatment, the membrane seems to reseal such that the treated cell should restore its initial physiological state. However, across different organisms, tissues, or cell types, nsPEFs were found to induce persistent changes of cellular state^1,2^. Likewise, nsPEFs were found to interfere with biological signaling^3,4^. In suspension cells of tobacco, nsPEFs modulated the activity a plant-specific kinesin motor at the cell membrane leading to specific responses of expansion axiality^5^, and in *Chlamydomonas reinhardtii* triggered oxidative burst and the formation of palmella stages^6^. In mammalian cells, nsPEFs treatment activated caspase activity and apoptosis^7,8^, which opens interesting applications for tumour and cancer therapy^9,10^. Further potential applications of nsPEFs include growth stimulation in plants^11^, boosting the yield of edible mushrooms^12^, the chemical free processing and preservation of food (for review, see^13^). For microalgae, they have been found to induce an increase in dry weight^14,15^ and even to trigger oxidative burst and palmella stage formation in *C. reinhardtii*^6^.

The work from mammalian cells suggests that nsPEFs modify intracellular targets in the first place, which is usually explained by the extremely short rise time that will unfold the effects of the field, before the plasma membrane had the chance to be fully charged^3^. However, there is clear evidence from plant cells for direct responses of the cytoskeleton at the plasma membrane^16^.

This diversity of cellular responses as well as the relatively low input energies that are usually far too small to electroporate the membrane (irreversibly) already indicates that a merely physical interpretation will not suffice to understand the effect of nsPEFs. Alternative working models approach focus on the modulation of signaling events at the cell membrane that might be altered even by electrical fields of moderate energies. For plants, two major signaling inputs are central: Reactive oxygen species (ROS) generated by the NADPH oxidase Respiration burst oxidase Homolog (RboH) located on the plasma membrane (reviewed in^17^), and the opening of voltage-gated calcium channels in the plasma membrane (reviewed in^18^) as central components of early stress signaling.

As photosynthetic life forms, plants must cope with a specific form of abiotic stress that occurs, when the electron-transport chain in the chloroplast becomes saturated at high light intensity. Excess electrons can be transferred to oxygen, producing the highly reactive superoxide O_2_^-^ and also cause auto-oxidation of chlorophyll. Carotenoids have evolved as accessory pigments that act as powerful anti-oxidants to protect photosynthesis against high-irradiance^19^. Among the numerous types of carotenoids found in different plants, astaxanthin (3, 3′-dihydroxy-β, β’-carotene-4, 4′-dione) is particularly powerful with respect to anti-oxidant activity able to neutralise free radicals as well as to quench ROS that are produced in response to environment stimulation^20^. Traditionally, astaxanthin has been used as food colorant, and as feed supplement for salmon and shrimp aquaculture^21^ (for review, see^22^). Meanwhile, the powerful antioxidant properties of astaxanthin have also attracted a strong interest in the context of nutraceutical prevention of inflammatory, or cardiovascular diseases, but also for the prophylaxis of cancerogenesis, and the stimulation of immune responses^23–26^. Astaxanthin can be synthetised by only a limited number of organisms, including a few bacteria (*Agrobacterium aurantiacum*^27^; *Paracoccus carotinifaciens* sp. nov.^28^), yeast (*Phaffia rhodozyma*^29^), plants^30,31^, and several green algae^32–36^. So far, the unicellular alga *Haematococcus pluvialis* shows the best potential to produce astaxanthin, accumulating up to about 4% of dry weight^37^.

**Figure 1:**
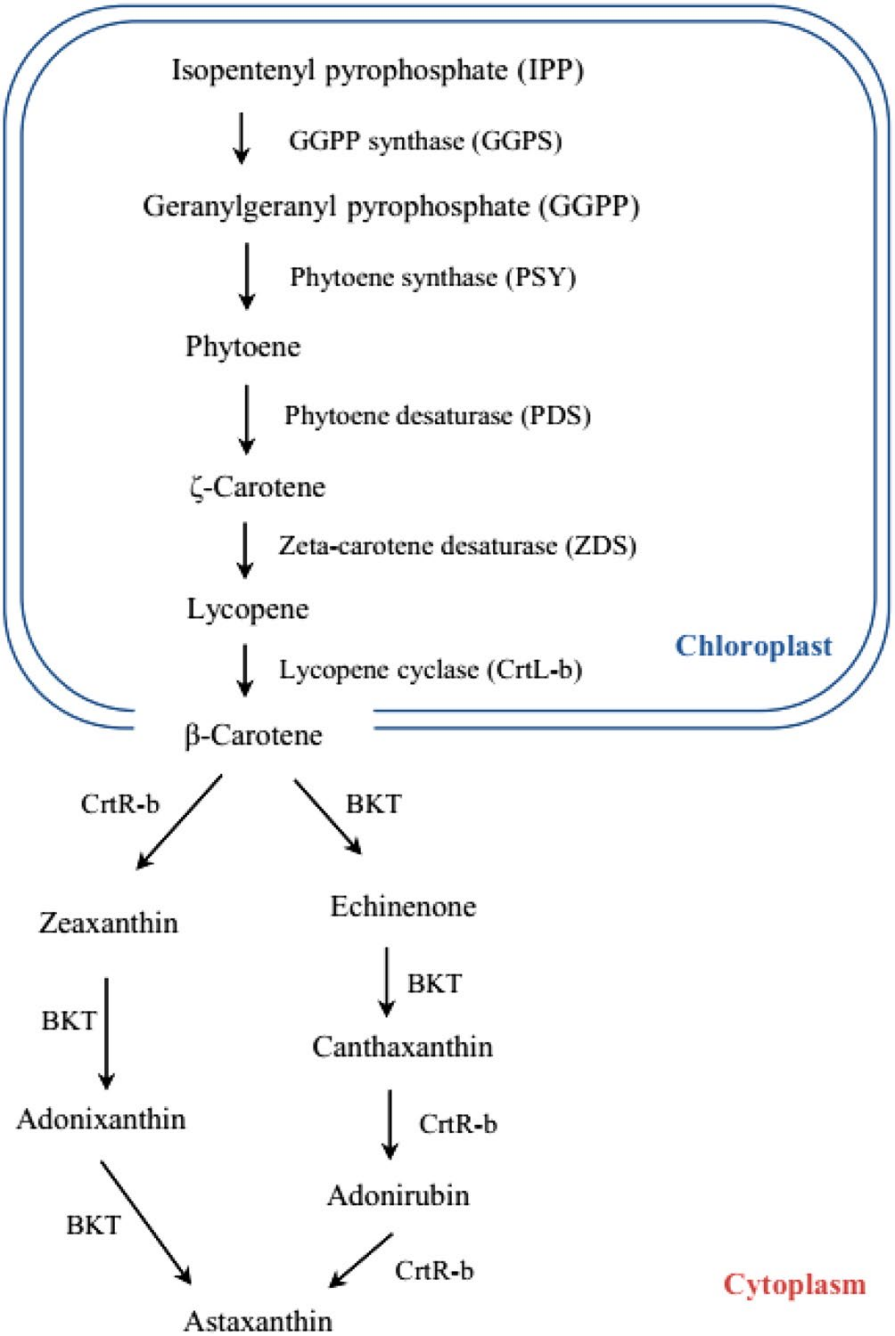
Astaxanthin biosynthesis pathway in the chloroplast and cytoplasm of *H. pluvialis* (modified according to^42^).

The growth of *H. pluvialis* can be divided into two stages: Under favourable conditions, cells are pear-shaped with a diameter ranging from 8 to 50 μm, endowed with two flagella, a cup-shaped green chloroplast, and an anterior eyespot^38^. However, in response to stress conditions, such as excessive light intensity, nutrient deficiency (nitrogen or phosphate), high salinity, or high temperatures^39–41^, the cellular morphology changes conspicuously: the cells round up and shed their flagella. Simultaneously, the cells swell and form a thick, resistant cell wall, while cell division is arrested^42^. After shifting into this non-motile encystment stage (termed palmella) astaxanthin accumulation initiates. Astaxanthin is synthesised in the plastid, but stored in cytoplasmic lipid vesicles^38,43^. As for other carotenoids, the synthetic pathway initiates in the chloroplast, using geranyl–geranyl pyrophosphate (GGPP) as the basic building block (Fig. 1). This building block is assembled by condensation through phytoene synthase (PSY) into the still colourless carotenoid phytoene. Subsequently, by successive desaturation reactions of phytoene, catalysed by phytoene desaturase (PDS) and zeta-carotene desaturase (ZDS), lycopene is formed. A subsequent cyclisation step by lycopene cyclase (CrtL-b) leads to β-carotene, which is then exported from the chloroplast into the cytoplasm, where the final steps of astaxanthin synthesis proceed (for review, see^42^). For the conversion of β-carotene into astaxanthin in *H. pluvialis*, two putative pathways have been proposed: either β-carotene is converted by BKT into canthaxanthin, which is then hydroxylated by β-carotene hydroxylase (crtR-b), or zeaxanthin is formed from the exported β-carotene by hydroxylation (mediated by crtR-b), and then the product subsequently undergoes oxidation by BKT (for review, see^42^). At last, the unstable free astaxanthin moiety is linked to fatty acids through an ester bond and stored in lipid vesicles, thus preserving the antioxidant activity by the lipophilic environment^43–45^. Irrespective of the proposed sequence of action, β-carotene hydroxylase (crtR-b) and β-carotene ketolase (BKT) are considered as the key enzymes for the conversion of β-carotene into astaxanthin, and, therefore, have been investigated on the molecular level^46,47^. For both, crtR-b and BKT, steady-state transcript levels have been reported to be induced under stress conditions^48,49^. The subcellular localisation is not totally clear: while an export of β-carotene from the chloroplast has been proposed^42^, a N-terminal plastidic signal peptide of 57 amino acid residues is predicted by TargetP (https://www.cbs.dtu.dk/services/TargetP/). For BKT, no putative signal peptide motif is detectable, while several predicted transmembrane proteins would link this protein rather into the ER membrane, which would also be compatible with the fact that astaxanthin is stored in oleosomes^50^.

In our previous work^6^, we had shown for *C. reinhardtii* (coming from a sister family of the same order within the Green Algae) that nsPEFs activated oxidative burst through the membrane located NADPH oxidase RboH, followed by palmella formation as specific developmental response. We, therefore, wondered, whether the same approach could be used to induce astaxanthin synthesis in *H. pluvialis*, and specifically, whether we can use this system to steer gene regulation by nsPEFs. As experimental model, we treated suspension cells of *H. pluvialis* with either a low (25 ns, 2 J·g^−1^), or an intermediate (50 ns, 4 J·g^−1^) dose of nsPEFs, and scored long-term cellular responses including astaxanthin accumulation along with the relative transcript level of the three key genes *bkt 1*, *crtR-b* and *psy*. Since *H. pluvialis* grows in a different pace from *C. reinhardtii*, the time-points of nsPEFs treatment and response analysis had to be adjusted accordingly. We observed that nsPEFs can modulate steady-state transcript levels in a pattern that was specific for the respective gene and the stringency of the PEF treatment. To determine the role of membrane located oxidative burst and calcium influx in the modulation of astaxanthin biosynthesis in the response to nsPEFs, we used the specific inhibitor diphenylene iodonium (DPI) to block the membrane located NADPH oxidase RboH, while cytosolic calcium levels were modulated either by the calcium ionophore A23187, or the calcium-channel inhibitor gadolinium (III) chloride (GdCl_3_). We found that the response of astaxanthin biosynthesis transcripts to nsPEFs depends on both, cytosolic calcium levels as well as RboH activity (giving rise to signaling ROS), whereby the response patterns differ for the individual genes indicative of a regulatory network targeted to different steps of the astaxanthin metabolic pathway.

## Results

### Modelling the effect of nsPEFs on membrane permeabilisation in *H. pluvialis*

To get insight into the long-term response of *H. pluvialis* to nsPEFs, cells were treated, at the end of proliferation (day 5 after subcultivation) with either moderate (25 ns, 2 J·g^−1^), or stringent (50 ns, 4 J·g^−1^) pulses, respectively.

**Figure 2:**
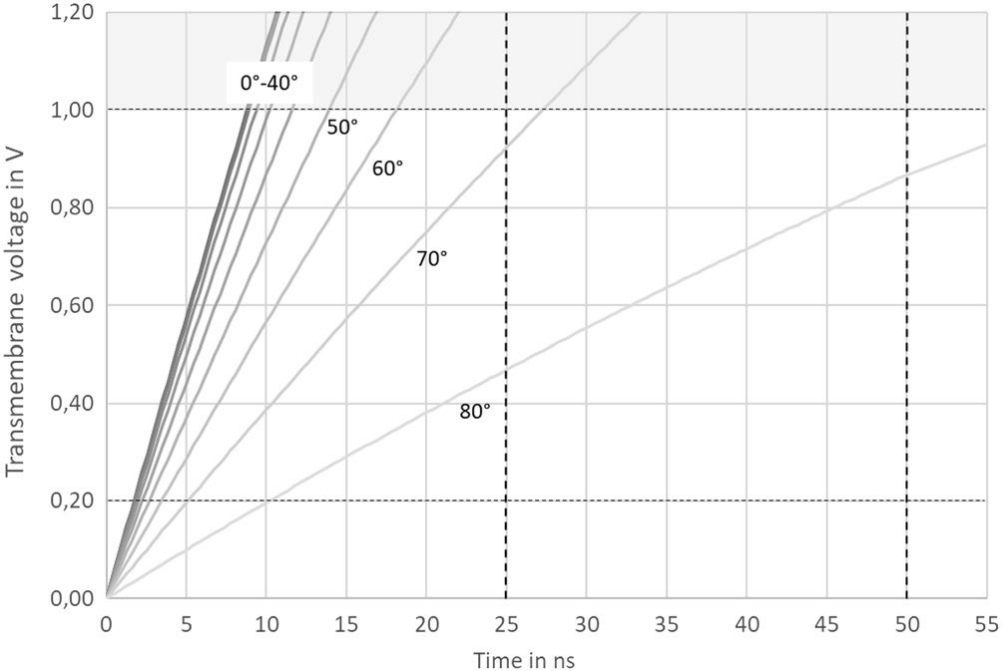
Modelling of transmembrane voltage (V_m_) over pulse duration at different azimuth with respect to the direction of the field for the conditions used in this study. Conductivity of the outer medium σ_e_ = 1.6 mS cm^−1^, estimated conductivity of the cytoplasm σ_i_ = 10 mS^.^cm^−1^, external electric field intensity E = 40 kV cm^−1^, specific membrane capacity C_m_ = 1 µF cm^−1^, spherical cell shape with radius a = 14 µm, time constant for cell polarization τ_C_ = 5.98·10^–7^ s under the assumption that membrane properties remain constant during the charging. Grey range (voltages > 1 V) are only hypothetical, but not relevant, because here, the voltage should break down due to membrane electroporation.

The modelling (Fig. 2) shows that the threshold for irreversible membrane electroporation (around 1 V) should be reached within 25 ns over around 2/3 of the entire circumference of the charged cell, while treatment with 50 ns should achieve the conditions for irreversible electroporation almost over the entire sphere (up to 80° azimuth angle). During the polarisation and electroporation of the outer membrane, the chloroplast membrane (which in *H. pluvialis* is very close to the plasma membrane, because the chloroplast fills almost the entire cell interior) can be permeabilised as well. For stringent treatment (with 50 ns), there should be sufficient time for substantial permeabilisation of the chloroplast, while for moderate treatment (25 ns), only initial steps of such a permeabilisation are expected. Although a strict separation of cellular effects for the two pulse treatments cannot be expected, it is safe to assume that the stringent treatment (50 ns) will not only permeabilise the plasma membrane, but the chloroplast as well.

### Long-term cellular response of *H. pluvialis* to nsPEFs

**Figure 3:**
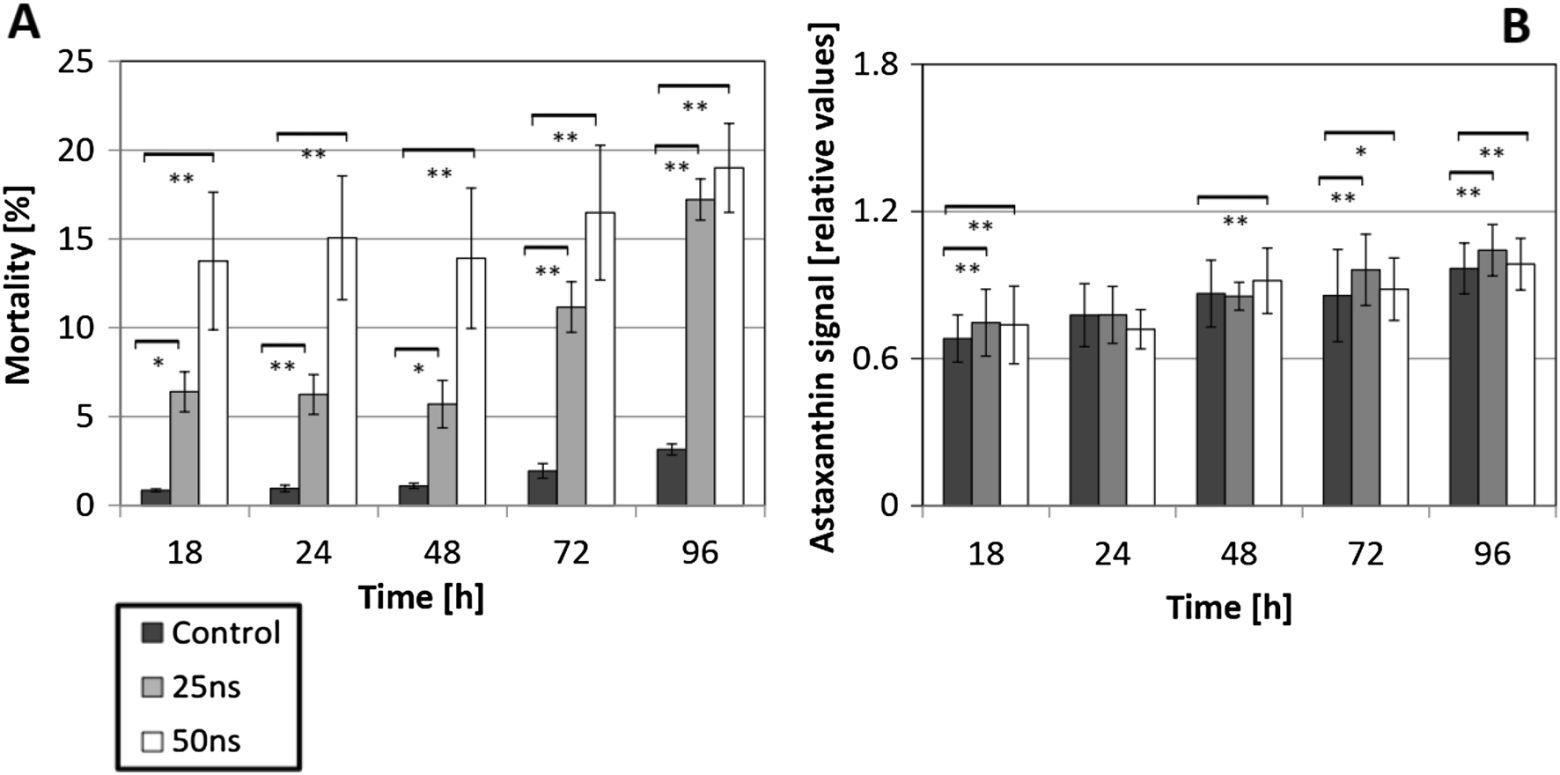
Responses of *H. pluvialis* to nsPEFs treatment. (**A**) Mortality measured as percentage of cells taking up the membrane-impermeable dye Evans Blue added at the respective time after PEF treatment. (**B**) Mean relative astaxanthin content quantified for individual cells by quantitative image analysis. Data represent mean values, error bars s_e_ of three independent experimental replications. Brackets indicate significant differences (*P < 0.05, **P < 0.01).

To validate the results from the modelling, mortality and astaxanthin content were determined at 18, 24, 48, 72 and 96 h after moderate (25 ns) and stringent (50 ns) PEF treatment (Fig. 3).

Mortality was measured based on the exclusion of membrane-impermeable dye Evans Blue by viable cells. In the control, mortality was below 3% during the whole experiment. In contrast, nsPEFs induced a significant increase of mortality (Fig. 3A). For instance, stringent treatment (50 ns) produced a mortality reaching up to 14% at 18 h after pulsing, which was about twice the value observed for moderate treatment (25 ns). Subsequently, for the stringent treatment (50 ns), mortality increased only slowly reaching about 19% at 96 h, while for moderate treatment (25 ns), mortality initially did not change and remained at about 6% until 48 h, but increased sharply from 72 h, reaching up to 17% at 96 h. Thus, nsPEFs treatment causes a significant increase in mortality from 3 to almost 20%. For a stringent pulse treatment (50 ns), this increase occurs rapidly and is almost complete at the first measured time point (18 h after the pulse), while for a moderate pulse treatment (25 ns), this increase occurs much later (from 72 h after the pulse). The astaxanthin signal increased moderately (by 20–30% of the initial value) over time (Fig. 3B) under all conditions. Although PEF treatment with 25 ns caused a slight, but significant increase for most time points (whereas pulsing with 50 ns was less effective in this respect), this increase was very small (in the range of 10% or less). Thus, the increase of mortality by nsPEFs treatment was not accompanied by any comparable increase in the accumulation of astaxanthin. Since astaxanthin content was determined for individual cells, based on quantitative image analysis, the marginal increase of astaxanthin content cannot be due to the increased mortality as it would be the case in a method, where astaxanthin accumulation would be measured over the entire population. Overall, on the level of the population, there seems to be a quite narrow band between the stimulation of astaxanthin, and impaired proliferation and viability of the cells.

### nsPEFs induce transcript accumulation for genes involved in astaxanthin biosynthesis

**Figure 4:**
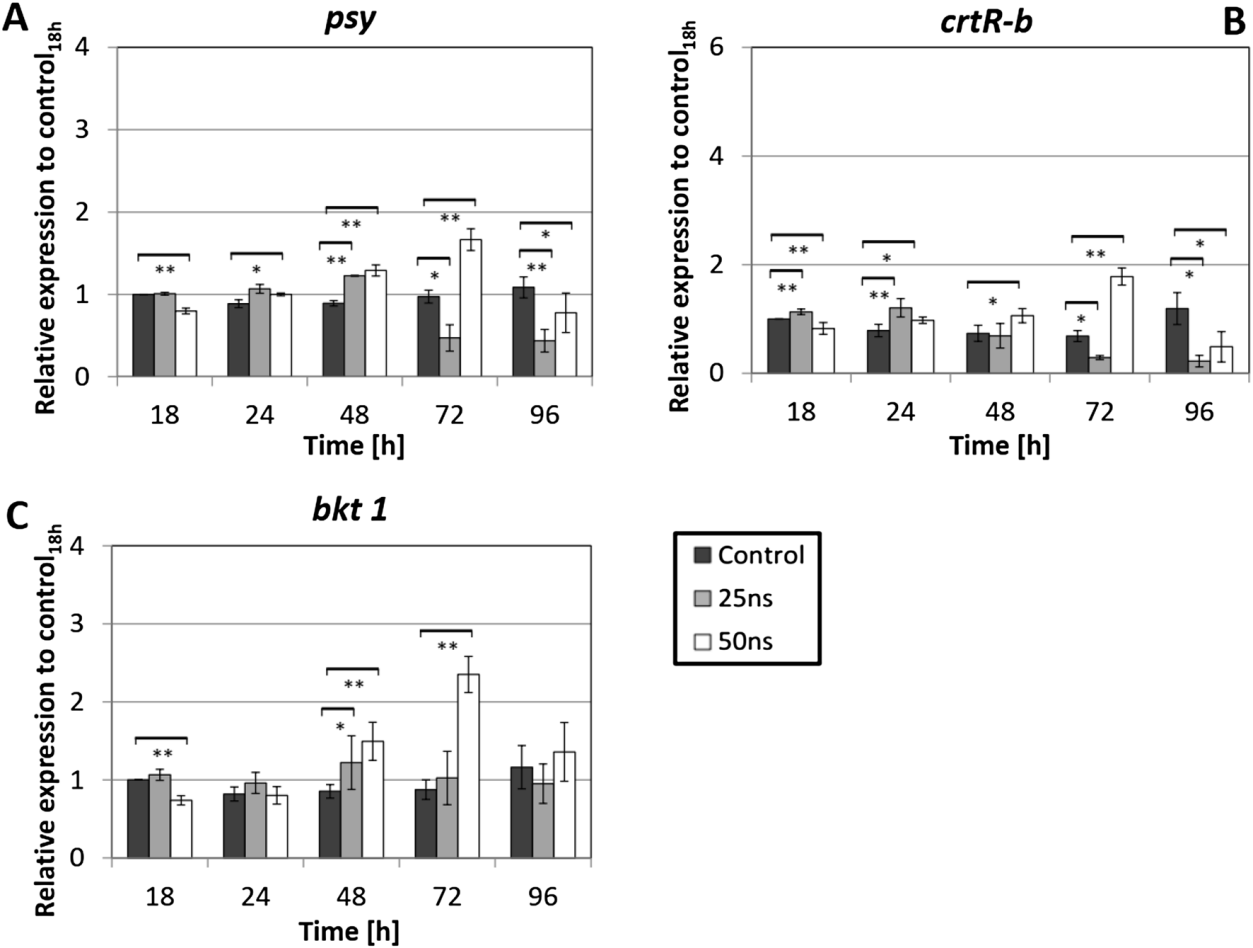
Responses of steady-state transcript levels for key genes of astaxanthin biosynthesis to nsPEFs treatment. (**A**) Phytoene synthase (*psy*). (**B**) β-carotene hydroxylase (*crtR-b*). (**C**) β-carotene ketolase (*bkt 1*). Gene expression was quantified against actin as housekeeping gene. Values are given relative to the control value measured at 18 h. Data represent mean values, error bars s_e_ of three independent biological replicates with three technical replicates each. Brackets indicate significant differences (*P < 0.05, **P < 0.01). Any difference not marked by brackets are not significant.

Since the abundance of astaxanthin responded only slightly to nsPEFs treatment (Fig. 3B), the biosynthetic pathway might simply not be responsive. Alternatively, the transcripts might accumulate, but their translation, the activity of the resulting proteins, or the availability of substrate might be limiting. To get insight into the status of the astaxanthin pathway, steady-state transcript levels were measured by qRT-PCR for the three key genes, phytoene synthase (*psy*), beta-carotene hydroxylase (*crtR-b*), and beta-carotene ketolase (*bkt 1*). Transcript levels for *psy* monitor the activity of the basal carotenoid pathway, while *crtR-b* and *bkt 1* are specific for the final part of astaxanthin biosynthesis (Fig. 4).

In fact, nsPEFs were able to modulate steady-state transcript levels for the three genes, but the pattern was dependent on the respective gene, and the stringency of the PEF treatment. To discern significant differences from the experimental noise that is not negligible in any quantitative study on gene expression, we conducted three independent experimental series with different culture batches and also did each measurement in three technical replicates. Thus, each data point represents a population of 3 × 3 individual transcript measurements. In the following, only those differences that are significant, will be described. For a stringent treatment (pulse duration 50 ns), all three genes responded in a similar manner: a slow increase from 48 h led to a peak of around twofold at 72 h (most pronounced in *bkt 1*), followed by a subsequent drop to around the initial level. For the moderate treatment (25 ns), the pattern was different: here, the transcript levels of *psy* and *crtR-b* decreased (most pronounced for *crtR-b* that dropped to around 1/3 of the initial level) from 72 h and did not recover, while the *bkt 1* transcripts remained basically unchanged.

Thus, although the level of the final product, astaxanthin, was not very responsive to nsPEFs treatment, there was a significant induction in the steady-state levels for transcripts of the astaxanthin pathway. Interestingly, while the pattern was similar for stringent treatment, a qualitative difference became manifest for moderate treatment, where *bkt 1* behaved differently from *psy* and *crtR-b*.

### Astaxanthin biosynthesis dependence on the activity of a membrane-located NADPH oxidase

**Figure 5:**
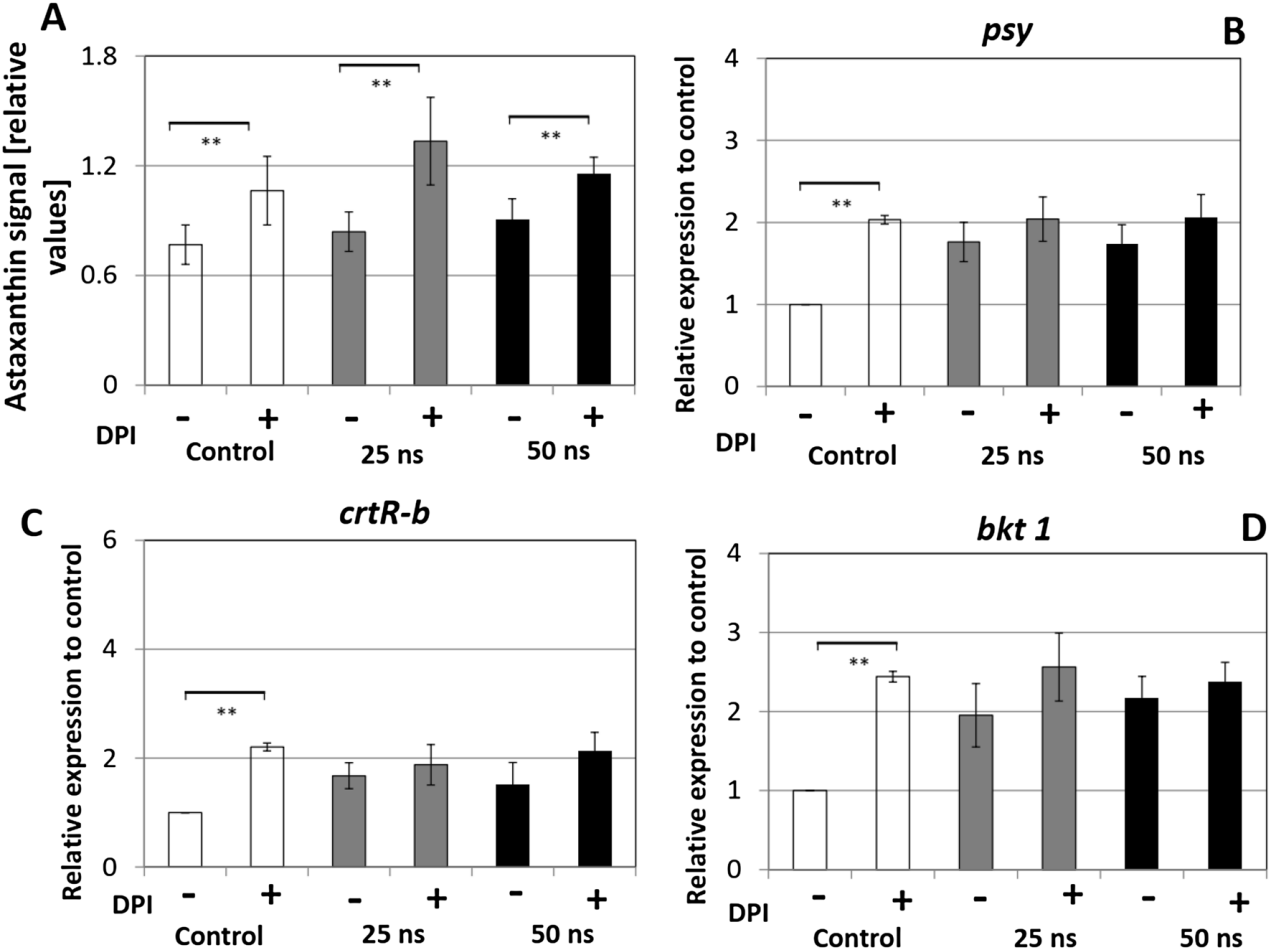
Effect of DPI (8 μM) as inhibitor of the NADPH oxidase RboH on the astaxanthin biosynthesis pathway. The inhibitor was added at 30 min before pulse treatment, and the measurements were recorded at 72 h after PEF treatment. (**A**) Astaxanthin content quantified for individual cells by quantitative image analysis. (**B**–**D**) Responses of steady-state transcript levels for (**B**) phytoene synthase (*psy*), (**C**) β-carotene hydroxylase (*crtR-b*), and (**D**) β-carotene ketolase (*bkt 1*). Gene expression was quantified against actin as housekeeping gene. Values are given relative to the control (without DPI). Data represent mean values, error bars s_e_ of three independent experimental replications. Brackets indicate significant differences (*P < 0.05, **P < 0.01). Any difference not marked by brackets are not significant.

In our previous work on nsPEFs responses in the microalga *C. reinhardtii*^6^, we had demonstrated that DPI, a specific inhibitor of the membrane-located NADPH oxidase RboH, suppressed the long-term responses to nsPEFs. We therefore asked, whether this NADPH oxidase modulates astaxanthin biosynthesis. DPI was added at 30 min before pulsing with durations of 25 ns and 50 ns, respectively. Then, the astaxanthin content and the relative gene expression of *psy*, *crtR-b* and *bkt 1* were quantified at 72 h after PEF treatment (Fig. 5).

To score the relative effect of DPI, the values were normalised to the value seen at 72 h for a sample neither pulsed, nor treated with inhibitor (note: the values cannot be compared to the time course in Fig. 4, which used a different internal standard), because, here, the effect of DPI is in the focus. We observed that astaxanthin content was significantly increased in response to DPI, for both control and nsPEFs treatment (Fig. 5A). This increase was most pronounced (+ 80%) for a pulse of moderate stringency treatment (25 ns), but weaker (+ 30%) for the non-pulsed control, as well as for the stringent pulsing (50 ns). The stimulating effect of DPI was also seen for the transcript level of the astaxanthin biosynthesis genes *psy*, *crtR-b* and *bkt 1* (Fig. 5B–D). For the non-pulsed controls, DPI treatment roughly doubled the levels of all three transcripts as compared to the control without DPI. For both pulse treatments, the transcript levels where already elevated in the absence of DPI (consistent with our previous experiment, Fig. 3). Here, the enhancement produced by DPI was less pronounced compared to a PEF treatment without DPI. Thus, the interaction of DPI and PEF treatment was not additive, but limited to a saturation level that was not exceeded.

### The role of Ca^2+^ influx for the nsPEFs response of astaxanthin biosynthesis

**Figure 6:**
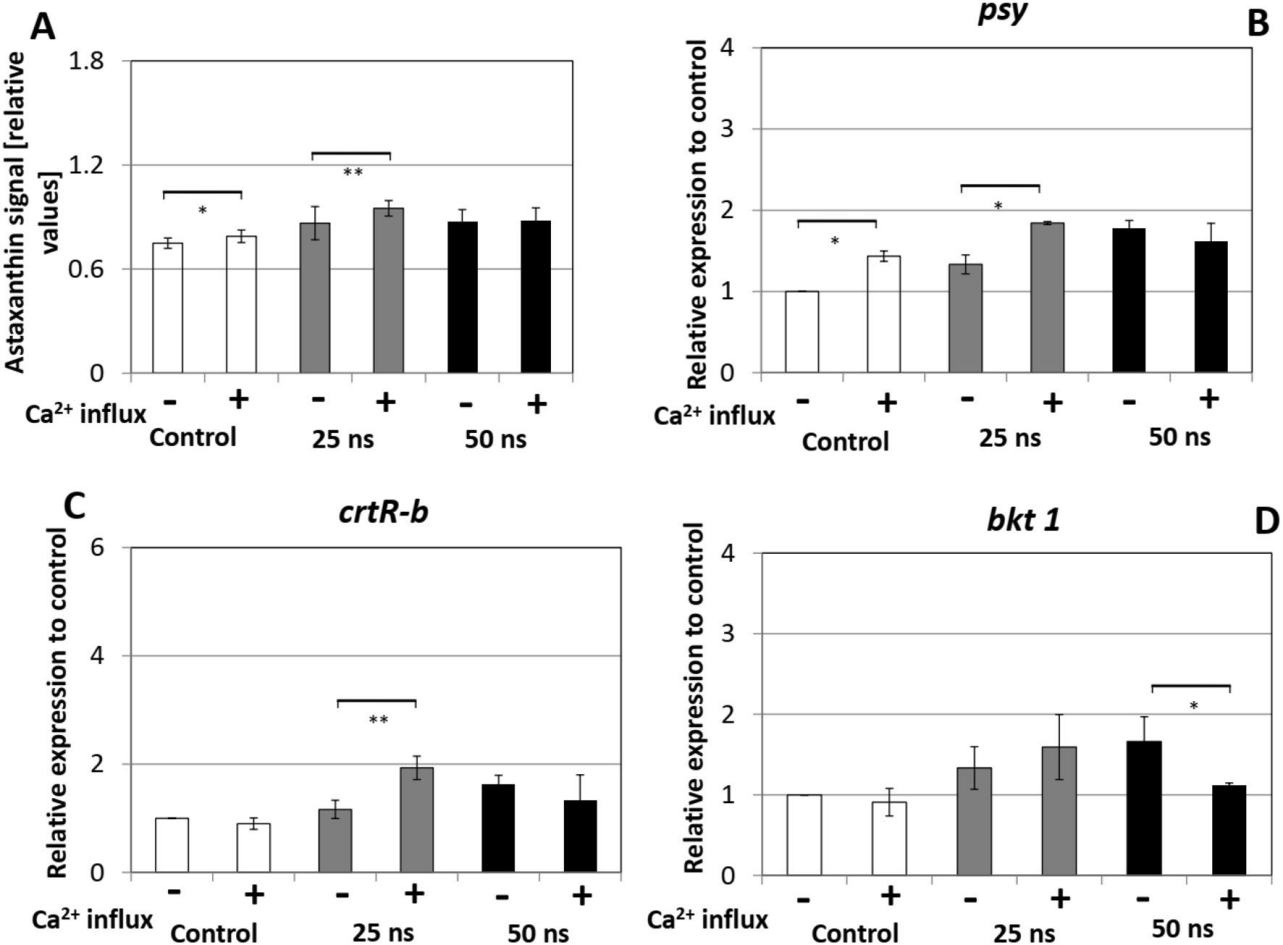
Effect of the calcium ionophore A23187 (40 μM) on the astaxanthin biosynthesis pathway. The ionophore was added at 30 min before the pulse treatment, and the measurements were recorded at 72 h after pulsing. (**A**) Astaxanthin content quantified for individual cells by quantitative image analysis. (**B**–**D**) Responses of steady-state transcript levels for (**B**) Phytoene synthase (*psy*), (**C**) β-carotene hydroxylase (*crtR-b*), and (**D**) β-carotene ketolase (*bkt 1*). Gene expression was quantified against actin as housekeeping gene. Values are given relative to the control (without ionophore). Data represent mean values, error bars s_e_ of three independent experimental replications. Brackets indicate significant differences (*P < 0.05, **P < 0.01). Any difference not marked by brackets are not significant.

**Figure 7:**
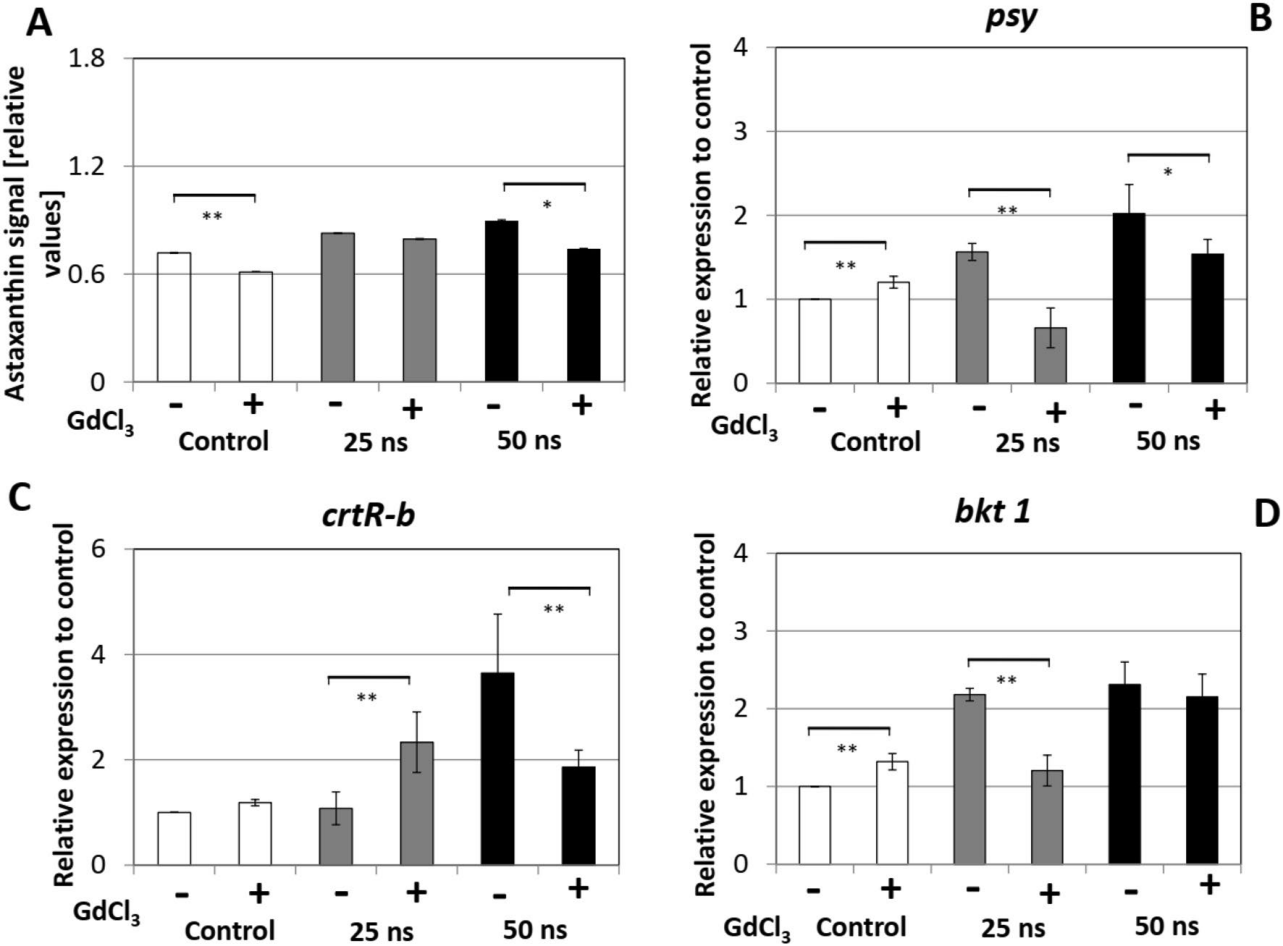
Effect of the calcium influx inhibitor GdCl_3_ (80 μM) on the astaxanthin biosynthesis pathway. The inhibitor was added at 30 min before the pulse treatment, and the measurements were recorded at 72 h after pulsing. (**A**) Astaxanthin content quantified for individual cells by quantitative image analysis. (**B**–**D**) Responses of steady-state transcript levels for (**B**) Phytoene synthase (*psy*), (**C**) β-carotene hydroxylase (*crtR-b*), and (**D**) β-carotene ketolase (*bkt 1*). Gene expression was quantified against actin as housekeeping gene. Values are given relative to the control (without GdCl_3_). Data represent mean values, error bars s_e_ of three independent experimental replications. Brackets indicate significant differences (*P < 0.05, **P < 0.01). Any difference not marked by brackets are not significant.

Calcium influx represents, together with RboH dependent apoplastic oxidative burst, a central input for plant-stress signaling. We therefore probed the role of Ca^2+^ for astaxanthin biosynthesis in response to nsPEFs stimulation using two complementary strategies: By using the calcium ionophore A23187 we asked, whether a calcium influx in absence of nsPEFs treatment would be sufficient to trigger the astaxanthin pathway, while GdCl_3_ used as Ca^2+^ influx inhibitor would test, to what extent calcium influx is necessary. Both inhibitors were added at 30 min before the pulsing treatment separately, and astaxanthin content and the transcript levels for *psy*, *crtR-b* and *bkt 1* were again measured at 72 h after pulsing (Figs. 6, 7).

When we increased the Ca^2+^ influx (by adding calcium ionophore A23187), this led only to a minor (but significant in some cases) increase in astaxanthin accumulation in the non-pulsed controls, and in the cells pulsed at 25 ns (Fig. 6). However, transcripts for *psy*, *crtR-b* and *bkt 1* showed a significant response to the ionophore (Fig. 6B–D). Calcium influx alone was sufficient to stimulate *psy*, but not the transcripts of the later astaxanthin pathway, nor the final product, astaxanthin. For pulsing with 25 ns, transcripts of *psy* and *crtR-b* were significantly elevated, but not those of *bkt 1*. In case of pulsing with 50 ns, the ionophore significantly reduced the response for *bkt 1* as compared to the value for 50 ns without ionophore.

To test, to what extent Ca^2+^ influx is necessary for the response of the astaxanthin pathway to nsPEFs, we tested GdCl_3_ as Ca^2+^ channel blocker. Here, we saw that astaxanthin accumulation decreased in the non-pulsed control as well as for nsPEFs-treated samples (Fig. 7A), which was more or less a mirror image of the situation seen with the ionophore (Fig. 6A). With respect to the transcripts of *psy*, *crtR-b* and *bkt 1* (Fig. 7B–D), the inhibition of Ca^2+^ influx (by adding GdCl_3_) more or less eliminated the stimulation of transcript levels by nsPEFs. This elimination was most pronounced for *crtR-b* (Fig. 7C), where the 50 ns pulse induced fourfold over the untreated control, while the same pulse after gadolinium pretreatment induced only twofold over the untreated control. There is one exception to the rule: this is the response of *crtR-b* to a moderate challenge with pulses of 25 ns duration. Here, GdCl_3_ stimulated this transcript, contrasting with the situation in the other two genes, and also contrasting with the situation for stringent treatment with durations of 50 ns. Thus, while transcripts of astaxanthin biosynthesis genes seemed to be modulated by calcium influx, this modulation differed partially even qualitatively, between the individual genes.

## Discussion

In our previous work, we had shown that nsPEFs can induce long-term developmental responses in microalgae, which was linked with activation of respiratory burst by the membrane-located NADPH oxidase RboH. In the current work, we extended the approach to the model alga *H. pluvialis*, and used nsPEFs to elicit specific changes in the expression of metabolic genes in order to modulate the accumulation of a valuable secondary compound, astaxanthin. We could show that nsPEFs treatment can induce significant changes in the expression of the astaxanthin biosynthesis genes *psy*, *crtR-b*, and *bkt1*, although the effect on astaxanthin accumulation was minor. To get insight into the early signals regulating these expression responses, we used pharmacological manipulation of two relevant signaling inputs that are targets for nsPEFs: the activity of the plasma-membrane located RboH (inhibited by DPI), and calcium influx at the plasma membrane (mimicked by the ionophore A23187, or inhibited by the calcium-channel blocker GdCl_3_). We can demonstrate that both signaling inputs are relevant for gene expression. Thus, this work provides a proof-of-concept that it is possible to use electro-manipulation in microalgae to modulate the expression of metabolic genes and to activate the accumulation of a value-giving compound.

Quantitative studies on gene expression have to handle experimental variation that is not negligible. To address this, we followed the standards of the field, which consists in three independent biological series with different culture batches, and three technical replicates per biological sample. This allows to discern biological differences from background noise. However, even if one filters out experimental variations focusing only on the statistically relevant difference, the data reveal a complexity that is considerable and that warrants explanation. The observed changes in steady-state transcript levels were not paralleled by a corresponding response of the final product, astaxanthin, which indicates that posttranscriptional regulation plays a significant role. Furthermore, the patterns seen for the expression of the three tested biosynthetic genes were specific, but complex and not only depend on the stringency of PEF treatment, but also on the particular gene. The same was observed for the regulatory impact of the two inputs (RboH, calcium influx). As we will show in the following, even non-intuitive aspects of the data set, such as the time-dependent differences in expression levels of the different transcripts can be understood as implications of a model, where two antagonistic signal pathways (retrograde signalling from plastid to nucleus, signalling from the plasma membrane) are activated differentially depending on the stringency of pulsing.

This working model is built upon three elements: (1), the differential effect of moderate (25 ns) versus stringent (50 ns) pulsing on plasma membrane versus inner membranes, (2), the different subcellular localisation of the three biosynthetic enzymes that is therefore under command of different regulatory circuits, and (3), the different role of the two signaling inputs, RboH and calcium influx, on these regulatory circuits.

(1) The different effects evoked by moderate versus stringent PEF treatment have to be discussed in the context of differential permeabilisation of the plasma membrane over internal membranes. Intracellular targets can be charged for extremely short rise times^3^. However, this cannot account for the difference between the two treatments in our study, since the rise times of both pulse treatments were identical. The plasma membrane of cells is a frequency dependent amplifier of the applied electromagnetic field E(f) and becomes electrically transparent at frequencies f >  ~ 100 kHz^51^. Accordingly, it has been argued that nsPEFs with frequency components f > 100 kHz (corresponding to pulse durations < 10 ns), provide a unique way to extend the reach of an applied electric field to intracellular structures, e.g., by altering the nuclear or mitochondrial transmembrane voltage. However, non-thermal intracellular effects, including organelle electroporation, can also be induced after or simultaneously with plasma membrane electroporation^52^, if one deals with pulse durations longer than few ns, at least > 25 ns as shown in the time course of the transmembrane voltage. Therefore, a straightforward assumption would be that the treatments induce pores in the plasma membrane. Principally, any field strength that is strong enough to induce aqueous pores into membrane by amplifying the innate aqueous in homogeneity that are present in any membrane. The size of the induced pores and the spread of permeabilisation over the azimuth of the pulsed cell is expected to grow with the duration of the pulse, while the incidence of pore formation depends on the intensity of the field. This means that higher field strength should induce more pores, while increasing pulse duration should lead to larger pores that also would allow better targeting of inner membranes. In fact, using Chinese Hamster Ovary (CHO) cells that have a similar size and geometry as *H. pluvialis* (around 10 µm, spherical shape), pulses of comparable dose (60 ns at 13 kV·cm^−1^) were shown to cause pore formation monitored by uptake of membrane-impermeable dyes^53^. Using the uptake of the drug bleomycin leading to cell death, a similar conclusion was obtained with pulses of 10 ns at 40 kV·cm^−1^, again in CHO cells^54^. Theoretical modelling predicts that inner membrane can be targeted also for longer pulses, once the plasma membrane has developed pores^52^. If these considerations are now applied to the current experiments, the following scenario seems feasible: The field strength was identical for both, the moderate and the stringent, pulse treatments. Thus, the number of pores is expected to be comparable for both treatments, while the pore size and also the spread of permeabilisation is expected to be larger for PEF treatment with 50 ns over that obtained for pulsing with 25 ns. Since *H. pluvialis* is endowed with a single, but very large chloroplast, the time to charge the chloroplast membranes is expected to be comparable to that needed to charge the plasma membrane. Thus, for PEF treatment at 25 ns, it is mainly the plasma membrane which is expected to be perforated, while for 50 ns, also the inner membrane should become permeabilised in addition. When a pattern of gene regulation is modulated differently, when the pulse duration is increased from 25 to 50 ns, this is most likely due to impaired tightness of chloroplast membranes.

(2) While all three genes investigated here, are encoded in the nuclear genome, the regulation of these three genes is expected to depend on their final subcellular localisation. Enzymes that are acting in the chloroplast, are translated at free ribosomes in the cytoplasm and then imported through Translocon at the Outer Chloroplast Membrane (TOC) and Translocon at the Inner Chloroplast Membrane (TIC) by virtue of a N-terminal signal peptide that is cleaved during this transport. This transport depends on the redox balance in the plastid^55^. However, also the expression of the respective genes depends on the plastidic redox balance, a phenomenon originally termed as plastid factor, meanwhile called retrograde signaling (reviewed in^56^). Among the three genes studied, the proteins encoded by *psy* and *crtR-b* are localised in the plastid (both of them carry N-terminal signal peptides predicted to confer plastid import), and therefore should be subject to retrograde signaling, while for the third gene, *bkt 1*, no plastid localisation motif could be detected, such that the expression of this gene should be independent of retrograde signaling. In fact, while for stringent pulsing, the three genes behaved in a similar manner, for moderate pulsing, *bkt 1* behaved differently from *psy* and *crtR-b*: the transcript levels of *psy* and *crtR-b* encoding proteins to be imported into the plastid decreased (most pronounced for *crtR-b*), while the *bkt 1* transcripts did not. This might, at first sight, support a scenario, where also at 25 ns, the plastid is permeabilised. However, it should be kept in mind that the genes are all part of the nuclear genome, such that they should be affected by intracellular fields in the same manner. Differences in activation must, therefore, involve some kind of specific signalling: Following the line of argument in (1), the moderate PEF treatment should induce signaling at the plasma membrane, while retrograde signaling from the plastid to the nucleus would still convey the information of intact redox balance at the plastidic electron transport chain, providing a negative feedback for the transcription of *psy* and *crtR-b*. For the stringent PEF treatment, also plastidic membranes are expected to lose their tightness, such that the proton gradient from the thylakoid lumen into the stroma should be impaired leading to a reduced accumulation of ATP. This would affect the efficiency by which the electrons from water splitting at photosystem II are channeled into the electron transport chain towards photosystem I. As a result, ROS, such as superoxide, accumulate^57^, which will deploy redox stress signaling to the nucleus^58^. The difference between *bkt 1* and the other two transcripts (Fig. 4) is expected to disappear when this signalling is disrupted by DPI, Gd^3+^, or the calcium ionophore. In fact, when the pulses were administered in presence of these inhibitors the regulatory patterns of the three transcripts became be equalised (Figs. 5, 6, 7).

(3) Since astaxanthin is probably acting as powerful antioxidant in redox control, the genes of the astaxanthin biosynthesis pathway are expected to be controlled by stress signaling. At least two major inputs for plant-stress signaling have been shown to be modulated by nsPEFs: one input is the NADH-oxidase RboH located in the plasma membrane^6^, the other input is the influx of calcium^59^. To address the role of RboH, we used the specific inhibitor diphenylene iodonium (DPI) and found that all three biosynthetic genes were upregulated, no matter, whether the respective gene product was localised in the plastid or not (indicating that retrograde signaling is not relevant for this pathway). Thus, the signaling deployed by ROS generated through by RboH acts as negative regulator of gene expression. By nsPEFs, this effect can be even enhanced, but not much and not in a manner that is additive. Furthermore, there is no difference between pulsing at 25 ns or at 50 ns, which is a further hint that a contribution from the plastid is not relevant for this ROS-dependent signal. The patterns obtained for the calcium ionophore A23187 differ a bit. Here, for the moderate pulse (25 ns), the amplification of the presumed increase in cytosolic calcium by the ionophore caused a significant induction for the two genes encoding plastidic enzymes (*psy, crtR-b*), but not for the enzyme located in the ER (*bkt 1*). The values reached for moderate pulsing in combination with the ionophore were equivalent to those seen for stringent pulsing (50 ns) without ionophore. If the ionophore was added here, this was not leading to a further promotion, but rather to a reduction, albeit not significant. The most straightforward explanation would be that calcium-dependent signaling acts positively upon gene expression, whereby this signal is most relevant for the two genes encoding the plastid localised enzymes which would indicate interaction of calcium with the retrograde signal from the plastid into the nucleus. The presumed permeabilisation of the plastid membrane by pulsing at 50 ns might act in a similar manner by releasing calcium from the plastid. In combination with the ionophore, this effect might be saturated (or even super-optimal). A *caveat* at this point is necessary though: The use of an ionophore will change the charging of the membrane. Irrespective of this *caveat*, calcium influx by itself seems already sufficient to modulate expression of some transcripts (*psy*, for instance).

The finding that the calcium influx blocker Gd^3+^ can inhibit the induction of *psy* and *bkt 1*, would also support a scenario of calcium signaling as positive regulator of gene expression. For stringent pulsing at 50 ns, it is again the genes for the plastidic enzymes that are inhibited most significantly, while *bkt 1* is not responsive, which is a counter image of the pattern seen with the calcium ionophore, and would lend further support to an interaction between retrograde and calcium-dependent signaling. Unexpectedly, *crtR-b* is induced in presence of Gd^3+^ at moderate pulsing (25 ns), which is difficult to explain on the base of calcium alone, but can be explained by interaction with oxidative signaling as will be pointed out below. Since Gd^3+^ cannot permeate through the plasma membrane, the calcium signal acting here must derive from calcium influx, not from calcium release from internal stores. However, similar to the ionophore, one has to keep in mind that Gd^3+^ is also affecting the membrane permeabilisation itself^60,61^, such that the observed effects might are likely to be influenced by modulation of the input.

**Figure 8:**
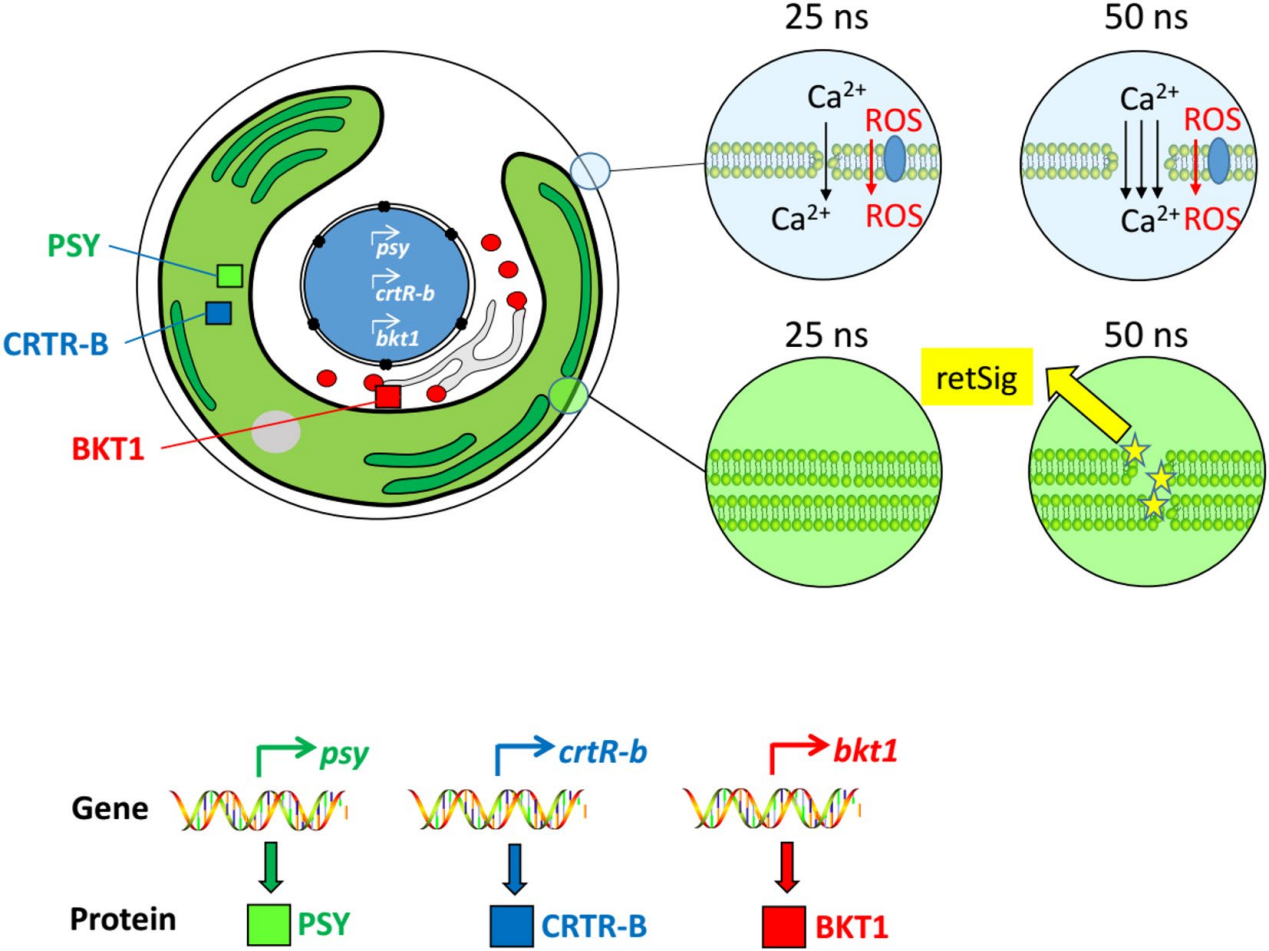
Working model of nsPEFs on *H. pluvialis*.

Thus, the observed, admittedly complex, pattern of regulation can be explained by a working model based on four elements (Fig. 8): (A) differential permeabilisation of inner membranes by moderate and stringent pulsing (in addition to the plasma membrane, which is permeabilised by both treatments), (B) retrograde signaling for genes of plastid-localised enzymes, (C) negative regulation of gene expression by RboH-dependent ROS signaling, which is independent of retrograde signaling, and (D) positive regulation of gene expression for the plastid-localised enzymes by calcium-dependent signalling that is dependent on retrograde signalling.

Using astaxanthin synthesis in *H. pluvialis* as experimental model, the current study provides a proof of concept that nsPEFs can be used to modulate expression of metabolic genes in a specific way. This specificity seems to result from differential permeabilisation of plasma versus chloroplast membranes, and different regulatory pathways for genes encoding proteins localised inside the plastid from those that act outside of the plastid. These findings open new avenues for electromanipulation of algal cells—by adjusting PEF treatment parameters, it should become possible to steer metabolic processes in algae for biotechnological applications by merely physical factors in a manner that is minimal invasive, reversible, and free of noxious chemical residues. To rephrase Richard Feynman’s famous statement given in Pasadena 1959: *There is plenty of room at the bottom (of electroporation)*.

## Methods

### Cell cultivation

The strain *H. pluvialis* SAG 192.80, obtained from the Culture Collection of Algae at Göttingen University, Germany, was used. Cells were grown in 40 ml of sterilised basal medium (pH 6.8)^62^ in a 100-ml Erlenmeyer flask under continuous white light (L 36 W/965; OSRAM, Germany) at a fluence rate of 25 μmol m^−2^ s^−1^ photosynthetically available radiation. The cell suspensions were maintained at 25 °C and manually shaken once per day. To standardise culture growth, an inoculum of stationary cells was diluted to an optical density (OD) of 0.1. The cultivation cycle was 7 days. *H. pluvialis* cells were streaked on the basal medium solidified by 2% (w/v) agar (gel strength: 950–1,050 g·cm^−2^) and subcultured every 30 days for backup.

### Nanosecond pulsed electric fields (nsPEFs)

Aliquots of 40 ml of *H. pluvialis* suspension were used for an individual nsPEFs experiment. The details of the treatment chamber and pulsed generator system were described by Eing et al.^11^, and the nsPEFs parameters used through our study were adopted from our previous study^6^ as follows:

The cell suspension flowed through a sterilised silica rubber tube driven by a peristaltic pump (Ismatec Ism 834C, Switzerland) from the bottom of the treatment chamber at a constant flow rate of 3.75 ml min^−1^. After passing through the treatment chamber (2 mm electrode gap), the cell suspension was collected in another empty, sterilized 100 ml Erlenmeyer flask sealed by a silicone plug covered by aluminum foil. In our study, a transmission line pulse generator delivered rectangular pulses with a voltage amplitude of 8 kV, which corresponds to 40 kV·cm^−1^ of the electric field strength. The frequency of the pulses was 4 Hz, and the conductivity of the basal medium was 1.6 mS·cm^−1^. The pulse durations were 25 ns and 50 ns, with each cell was in average experiencing 32 pulses during the passage through the treatment chamber (Table 1), corresponding to 2 J·g^−1^ and 4 J·g^−1^ of specific input energy respectively. These parameters were derived from our previous study on *C. reinhardtii*^16^ and have been found to provide the right balance between maintaining viability and imposing a sufficient stimulus to lead to measurable readouts. Since *H. pluvialis* in terms of size and inner architecture is fairly close, the behaviour was expected to be comparable. In a previous study on plant cells^11^, we had tested different combinations of pulse duration and field strength and found that reciprocity was valid for short pulse durations, but was lost when the pulse is exceeding 50 ns. The waveform of the pulse electric field is shown in the Supplementary Fig. S1 for both durations, 25 ns and 50 ns.

**Table 1:** Basic parameters of nsPEFs.

	Field strength (kV^.^cm^−1^)	Pulsed duration (ns)	Conductivity (mS cm^−1^)	Pump flow rate ( ml min^−1^)	Frequency (Hz)	Number of pulses	Specific treatment energy (J g^−1^)
Parameter 1	40	25	1.6	3.75	4	32	2
Parameter 2	40	50	1.6	3.75	4	32	4

Membrane charging of spherical cells (diameter: 2a) by an external electric field E results in a sinusoidal azimuthal distribution of the transmembrane voltage Vm, which is given by^63^ and shown in Eqs. (1) and (2):

1 $${V}_{m}\left(\theta ,t\right)=Eacos(\theta )\bullet \left(1-{e}^{- \frac{t}{{\tau }_{C}}}\right)$$

2 $${\tau }_{C}=a{C}_{m}\left(\frac{1}{{\sigma }_{1}}+\frac{1}{{2\cdot \sigma }_{e}}\right)$$

C_m_ designates the membrane capacity per area, σ_i_ and σ_e_ intracellular and external medium conductivity. Membrane voltage exponentially rises with a time constant of τ_c_, when a fast rise time rectangular field pulse (step function) is applied to the cell suspension. The characteristic frequency of the β-dispersion of the cell suspension is than fc = 1/τ_c_ (in the range from 10 to 100 MHz). The applied pulse durations are in the same order as the time charging constant of the plasma membrane.

In this work, we investigated, whether nsPEFs can trigger astaxanthin biosynthesis in *H. pluvialis*, and whether ROS and calcium as important signaling molecules induced by nsPEFs will modulate astaxanthin biosynthesis.

To verify, whether nsPEFs can trigger the biosynthesis of astaxanthin in *H. pluvialis*, suspensions were treated 5 d after subcultivation with nsPEFs. This time point was selected based on preparatory experiments, where astaxanthin response and physiology had been followed over the culture cycle. At this time point, the cells have completed their proliferation phase, and astaxanthin starts to accumulate. After the pulse treatment, all samples were transferred back to the incubator, and cell mortality and astaxanthin content were measured at 18, 24, 48, 72 and 96 h after PEF treatment. To study the responses of gene expression involved in the astaxanthin biosynthesis to the stimulation by nsPEFs, the transcript level of three key genes were quantified by real-time qPCR: β-carotene ketolase (*bkt 1*), β-carotene hydroxylase (*crtR-b*), and phytoene synthase (*psy*). To assess the role of ROS and calcium influx during nsPEFs modulated astaxanthin biosynthesis, we used the specific inhibitor DPI, blocking the membrane located NADPH oxidase RboH, the calcium ionophore A23187, and the calcium-influx inhibitor GdCl_3_. The inhibitors were added in parallel experiments at 30 min before the PEF treatment, quantifying astaxanthin content and gene expression at 72 h after treatment.

### Mortality

To determine mortality, the Evans Blue Dye Exclusion Assay was used with minor modifications according to our previous work^6^: 2 ml of cell suspension were spun down in a 2-ml reaction tube (Eppendorf, Hamburg) at 4,000×*g* for 2 min (PICO 17, Thermo Scientific, Germany). Then, 1 ml of 2.5% Evans Blue (w/v, dissolved in double distilled water) was used to replace the supernatant. After staining for 5 min, Evans Blue was removed by centrifugation at 4,000×*g* for 1 min, and 1 ml of fresh basal medium was used to wash off the unbound dye. This washing step was repeated until the supernatant was colourless. Finally, the cells were concentrated in 200 μl of fresh basal medium, and 15 μl aliquots were scored for blue (dead) cells by means of a hematocytometer (Fuchs-Rosenthal) under an Axioskop microscope (Axioskop 2 FS; Zeiss, Germany) using a 20 × objective. Data represent mean values and standard errors of at least 1,500 cells per experiment and three independent experimental series.

### Quantification of astaxanthin content

Astaxanthin content was analysed by quantitative image analysis using the software Image J (https://imagej.nih.gov/) based on RGB images acquired by an Axioskop microscope (Axioskop 2 FS; Zeiss, Germany) with a digital camera (AxioCam color) and exported as “.tif” format with the AxioVision (Rel. 4.8) software. To quantify astaxanthin content, the RGB image was first inverted, and then split into green, red and blue channels. Under these conditions, the green channel represented the astaxanthin signal, while the red channel represented the chlorophyll signal. By subtracting the red channel from the green channel, the values for astaxanthin could be corrected against chlorophyll, such that the resulting signal corresponds to the abundance of astaxanthin in each cell. At last, the integrated density over each individual cell was quantified. The results represent mean values of at least 500 cells per each data point and standard errors of three independent experimental series.

### Inhibitor experiments

To investigate, whether the membrane located NADPH-oxidase RboH was involved in the signal transmission during the initiation of astaxanthin biosynthesis after nsPEFs stimulation, the RboH specific inhibitor DPI (8 μM, Sigma- Aldrich, Germany) was added at 30 min before nsPEFs treatment (at 5 d after subcultivation). During preparatory experiments, different concentrations of DPI had been tested, and 8 μM of DPI were selected, because at this concentration, significant inhibition was observed, while preserving viability. DPI was dissolved in 100% DMSO to prepare a 20 mM stock solution. As negative control, samples with the respective solvent in the same final concentration were prepared and processed in the same manner. To verify, whether calcium influx modulates the response to PEF treatment, the Calcium Ionophore A23187 (40 μM, Sigma-Aldrich, Germany), or the Ca^2+^ channel blocker GdCl_3_ (80 μM, Sigma-Aldrich, Germany) were added at 30 min before PEF treatment, respectively. The Ca^2+^ influx inhibitor A23187 was dissolved in 100% DMSO to prepare a 10 mM stock solution, while the Ca^2+^ channel blocker GdCl_3_ was dissolved in sterilized double distilled water and also to prepare a 10 mM stock solution. Solvent controls were conducted as described for DPI. After pulsing, all samples were transferred back to the incubator and cultivated for 72 h. Then, the astaxanthin content and the relative gene expression of *psy*, *crtR-b* and *bkt 1* were quantified for each nsPEFs treatment.

### RNA isolation and cDNA synthesis

Aliquots of 4 ml cell suspensions were harvested and the supernatant was removed after centrifugation at 10,000×*g* for 4 min. Then, samples were shock-frozen in liquid nitrogen immediately. One sterilized steel bead was added and the sample was homogenised twice by a Tissuelyser (Qiagen, Retsch, Germany) at 18 Hz for 30 s. Total RNA was isolated using the innuPREP Plant RNA Kit (analytic jena, Jena, Germany) according to the manufacturer protocol. RNA concentration was quantified photometrically (D30, Eppendorf, Germany).

The mRNA was reversely transcribed into cDNA by using the M-MuL V RTase cDNA Synthesis Kit (New England BioLabs; Frankfurt am Main, Germany) following the manufacturer instructions by a two-step reverse transcription protocol in a 20-μl reaction system, and carried out on a Primus 96 advanced PCR thermal cycler (PEQLAB Biotechnologie GmbH, Germany). During step one, 1 μg RNA as template were thoroughly mixed with 2 μl of Oligo dT, 1 μl of dNTPs (10 mM), and 13 μl of nuclease-free water. Then, the mixed sample was incubated at 70 °C for 5 min controlled by the PCR program. Subsequently, in step two 2 μl of 10 × Reverse Transcript (RT) buffer, 1 μl of RNAse inhibitor, and 1 μl of M-MulV RTase were thoroughly mixed into, and the sample transferred back to the PCR system and incubated at 42 °C for 1 h, and then at 90 °C for 10 min. Finally, the cDNA was diluted tenfold by nuclease-free water and stored at -20 °C till further analysis.

### Quantification of gene *psy*, *crtR-b* and *bkt 1*

To investigate the relative mRNA transcript levels of the genes *psy*, *crtR-b* and *bkt 1* in response to nsPEFs treatment, quantitative real-time PCR (qRT-PCR) was performed using the primers shown in Table 2. The qRT-PCR products were quantified by a CFX96 Touch Real-Time PCR Detection System (Bio-Rad, Germany), in 20-μl of a reaction system containing 4 μl of 1 × GoTaq colorless buffer, 11.75 μl of nuclease-free water, 0.4 μl of dNTPs (10 mM), 0.4 μl of each primer (10 mM), 1 μl of MgCl_2_ (50 mM), 0.1 μl of GoTaq polymerase (Promega, Mannheim, Germany), 0.95 μl 10 × SYBR green (Invitrogen, Darmstadt, Germany), and 1 μl of a 1:10 dilution of the cDNA template. The amplification conditions were 95 °C/3 min, followed by 39 cycles of 95 °C/15 s of denaturation, 63 °C/40 s of annealing and 72 °C/30 s for extension. As negative blank, reactions were amplified by using nuclease-free water instead of cDNA template. *Actin* was used as a housekeeping reference^64^, and the C_t_ values from each measurement were normalized to the respective value of *actin* as internal standard, such that the relative expression level of each gene could be normalized to the control value measured at 18 h. The final result was expressed as $2^{{ - \Delta \Delta {\text{C}}_{{\text{t}}} \left( {\text{X}} \right)}} .$

**Table 2:** Primers and literature references used for qRT-PCR.

Name	GenBank accession No	Primer sequence (5′–3′)	Reference
*psy*	AF305430	F: AGGGGATGCGGATGGATTTGC	^48^
		R: AGTCATCAGGCCAACAGTGCC	
*crtR-b*	AF162276	F: TGCTGCTACCACGATGCTGT	^65^
		R: CATGCAGGCAGACTTTGGGC	
*bkt 1*	GU143688	F: CACAGCTAGACGGATGCAGCT	^49^
		R: GCCTGCAACCTCCTTCTCCTT	
*actin*	DV203941	F: GCGGGACATCAAGGAGAAGCT	^64^
		R: TCGTAGTGCTTCTCCAGTGCC	

### Statistical analyses

All experiments were performed in three independent experimental series. The data represent mean values and standard errors. Significance of measured differences were tested by a t-test using Microsoft Office Excel Software (version 2010) for significance levels of *P < 0.05, or **P < 0.01.

## Supplementary information

Supplementary file1

## Data Availability

The datasets generated during and/or analysed during the current study are available from the first author on reasonable request.
