# Supplementary material for: Nanosecond pulsed electric fields modulate the expression of the astaxanthin biosynthesis genes psy, crtR-b and bkt 1 in Haematococcus pluvialis
Source: Sci Rep. 2020 Sep 23;10:15508. doi: 10.1038/s41598-020-72479-5 (PMC7511312; doi:10.1038/s41598-020-72479-5)
Supplement: Supplementary file 1 — Supplementary file1 [file 41598_2020_72479_MOESM1_ESM.pdf]

Nanosecond pulsed electric fields modulate the expression of the astaxanthin biosynthesis genes *psy*, *crtR-b* and *bkt 1* in *Haematococcus pluvialis*

Fan Bai [a], Christian Gusbeth [b], Wolfgang Frey [b], Peter Nick [a*]

[a] Botanical Institute, Karlsruhe Institute of Technology, Fritz-Haber-Weg 4, 76131 Karlsruhe, Germany

[b] Institute for Pulsed Power and Microwave Technology (IHM), Karlsruhe Institute of Technology, Campus Nord, 76344 Eggenstein-Leopoldshafen, Germany

* Corresponding author. Tel.: +49 721 608 42144 / 42142; fax +49 721 608 4193

E-mail addresses: baifan1215@aliyun.com (F. Bai), christian.gusbeth@kit.edu (C. Gusbeth), wolfgang.frey@kit.edu (W. Frey), peter.nick@kit.edu (P. Nick).

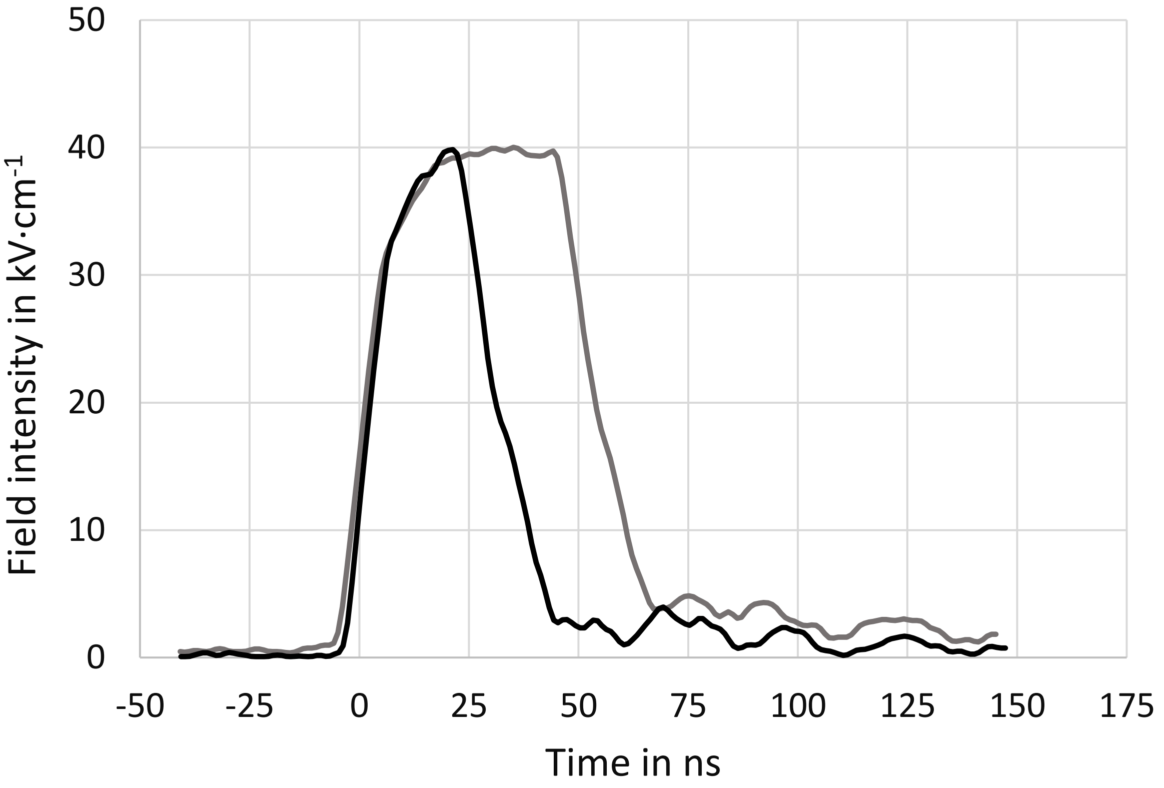

Supplementary figure S1. Waveform of the electric field pulse with a pulse duration of 25 ns (black) and 50 ns (grey). The displayed waveform is calculated from the voltage measurement across the electrode divided by the gap distance of 0.2 cm.
